# Mediastinitis caused by *Mycoplasma hominis* in immunocompetent patients: A case series report and literature review

**DOI:** 10.1016/j.heliyon.2024.e39763

**Published:** 2024-10-24

**Authors:** Fang Wang, Qing Zhan, Anfeng Yu, Hongchao Chen, Yan Zhang, Qing Yang, Tingting Qu

**Affiliations:** a Infection Control Department, The First Affiliated Hospital, Zhejiang University School of Medicine, Hangzhou, 310003, People's Republic of China; b Department of Cardiovascular Surgery, The First Affiliated Hospital, Zhejiang University School of Medicine, Hangzhou, 310003, People's Republic of China; c Department of Laboratory Medicine, The First Affiliated Hospital, Zhejiang University School of Medicine, Hangzhou, 310003, People's Republic of China; d State Key Laboratory for Diagnosis and Treatment of Infectious Diseases, National Clinical Research Center for Infectious Diseases, Collaborative Innovation Center for Diagnosis and Treatment of Infectious Diseases, The First Affiliated Hospital, Zhejiang University School of Medicine, Hangzhou, 310003, People's Republic of China

**Keywords:** *Mycoplasma hominis*, Mediastinitis, Omadacycline, Metagenomic next-generation sequencing

## Abstract

**Background:**

*Mycoplasma hominis*, a commensal organism, is potentially pathogenic; its role in postoperative infections might be underestimated in cardiac surgery.

**Results:**

We reported two cases of postoperative *M. hominis* mediastinitis in immunocompetent patients with a DeBakey grade I aortic dissecting aneurysm and reviewed 10 other cases previously described. Among the 10 reviewed cases and our two cases, 11 patients were men (median age, 59 years; median onset of clinical symptoms time, 14.5 d after surgery; and mean peak of temperature, 38.5 ± 0.8 °C). In our reports, two patients underwent sternotomy site reopening and debridement before diagnosis was confirmed. Diagnosis was confirmed by prolonged culture and by performing metagenomic next-generation sequencing directly using the clinical samples. *M. hominis* was difficult to cover with initial empirical antibiotic therapy; the patient in this study showed complete improvement with long-term antimicrobial therapy. The targeted treatment duration for surviving patients among the reviewed cases ranged from three weeks to 16 months.

**Conclusions:**

The diagnosis of extragenital *M. hominis* infections is difficult. Therefore, the role of *M. hominis* as a cause of postoperative infections during cardiac surgery should be considered. Diagnosis requires molecular techniques to complement culture.

## 1 Introduction

*Mycoplasma hominis (M. hominis),* belongs to the genus *Mycoplasma* of the family *Mycoplasmataceae,* in the class Mollicutes and order Mycoplasmatales. *M. hominis* was the first *Mycoplasma* isolate recovered from humans. Dienes and Edsall in the year 1937, isolated *M. hominis* from a Bartholin's gland abscess. They are coccoid in shape and much smaller than other bacteria, with a diameter of approximately 0.3 μm. *M. hominis* formed a typical fried-egg colony on agar. Prokaryotes that lack a cell wall frequently colonise the human genitourinary tract [1]. Their lipoproteins play a vital role in pathogenicity and are involved in a variety of functions, including adherence to host cells, cytotoxicity, immune evasion through antigenic variation, nutrient uptake, and direct interaction with the host's immune system [2]. In a laboratory, *M. hominis* is often missed because of the difficulty in detecting it using routine laboratory methods; however, this problem has recently been solved by applying molecular techniques [3]. *M. hominis* infections typically affect the genitourinary tract and cause urethritis, acute and chronic pyelonephritis, cervicitis, and pelvic inflammatory diseases. It rarely causes infection in immunocompetent adults, but can also cause serious extra-genital infections, generally in immunosuppressed or predisposed patients [4]. Moreover, infection with *M. hominis* may be more prevalent than previously indicated because its pathogenic action mechanisms at different levels can affect the joint (periprosthetic and septic arthritis), nervous system (drainage-related ventriculitis and neonatal meningitis), and even the cardiovascular system (mediastinitis and endocarditis). The route of infection is usually through urethral catheterisation or the respiratory tract, and predisposing factors for non-genitourinary infection include immunosuppression, trauma, or poor cardiorespiratory function [5].

Mediastinitis after cardiac surgery is a serious complication that can lead to high mortality rates even after aggressive intervention and antibiotic treatment. Previously uncommon pathogens such as *M. hominis* are no longer silenced in surgery-associated infections, especially since this pathogen is intrinsically resistant to antimicrobial agents such as β-lactams typically used in the perioperative period because this organism lacks a cell wall [1]. In addition, the increasing drug resistance of *M. hominis* to available drug options such as tetracycline and fluoroquinolones has resulted in delayed initiation of appropriate therapies and effects [6].

Here, we report two rare cases of postoperative mediastinitis caused by *M. hominis* after cardiac surgery in two immunocompetent adult patients and present a literature review of previous reports on mediastinitis caused by this fastidious microorganism.

## 2 Case series report

### 2.1 Case 1

A 71-year-old immunocompetent man with a medical history of hypertension for more than 10 years presented with chest and back paroxysmal tearing pain, which gradually worsened over 4 d. The patient was immediately transferred to the emergency department, and a DeBakey grade I aortic dissecting aneurysm was diagnosed by thoraco-abdominal aorta computed tomography (CT) angiography, with a haematocele around the ascending aorta root sides, mild pericardial effusion, and bilateral pleural effusion. Resistance occurred during the pre-operative insertion of the urinary catheter in this patient because of the narrow urinary tract and right renal calculi. The patient underwent emergency total aortic arch and ascending aorta surgical replacement and stented elephant trunk implantation. The operation was successful but was lengthy; cefuroxime sodium (3 g divided into two groups) was prophylactically administered during this stage, and drainage tubes were placed in the pericardium and mediastinum.

The postoperative course was initially favourable, and the patient's condition was stable. The empirical anti-infection medication cefuroxime sodium (1.5 g every 12 h) was administered. Routine urine tests revealed elevated levels of red blood cells and bacteria counts. On the 7th postoperative day (POD), the patient showed high inflammatory parameters (C-reactive protein 259.94 mg/L but procalcitonin (PCT) normal); cefuroxime sodium was stopped, and meropenem (1.0 g every 12 h) was introduced empirically. Simultaneously, the surgical wound was clean, with no signs of infection, and the patient remained feverless. However, on POD 14, clinical abnormalities manifested with persistent fever (maximum 38.2 °C) and an unstable sternum, which was confirmed by sternal palpation during ward rounds. Computed tomography (CT) showed increased pericardial and right pleural effusions. The patient underwent thoracic and pericardial puncture with catheter insertion, and drainage fluid samples were submitted for bacterial culture. Mediastinitis was suspected on POD 19. The patient underwent sternal wire removal, sternotomy site reopening, debridement, and vacuum-assisted closure. Large amounts of necrotic tissue formed in the mediastinal cavity. Aggressive surgical drainage and debridement of the infected tissue were performed, and drainage tubes were placed in the pericardium and mediastinum. Pericardial and pleural discharge cultures and matrix-assisted laser desorption/ionisation time-of-flight mass spectrometry (MALDI-TOF MS) were positive for *M. hominis* on the same day. To confirm this unusual result and investigate the possibility of a mixed infection, the clinician submitted a whole blood sample for metagenomic next-generation sequencing (mNGS), and it reported positive for *M. hominis*. Treatment was initiated with omadacycline (100 mg every day, doubling the initial dose) and meropenem (1.0 g every 8 h) after sternal debridement. The patient remained in a state of low fever (37.3–37.9 °C) in the following days, and the blood culture remained negative. On POD 26, a second debridement procedure was performed with dilute iodophor, local saline solution, and omadacycline (0.4 g) irrigation of the mediastinum; a large amount of new granulation tissue and less necrotic tissue in the mediastinal cavity was observed. The sternum was successfully closed using steel wires, and the drainage tubes remained intact after debridement. Given the favourable course of antibiotic treatment, omadacycline (100 mg daily) was continued. Cultures from effusion specimens collected 3 d after omadacycline treatment were negative for *M. hominis.* The drainage tubes were removed after a few weeks and the surgical wound healed well. On POD 78, the patient showed complete recovery and was discharged after 52 d of omadacycline antimicrobial therapy. Oral levofloxacin (oral 500 mg daily) was continued for 56 d after discharge. Table 1 shows the results of relevant blood tests during the hospital stay.

**Table 1:** Results of relevant blood investigations at infection onset, after 14 days of antibiotic treatment and the day before discharge.

Variable	Results			
	Reference range in our hospital	The point of infection onset	14 days after targeted treatment	The day before discharge
Case 1				
Body temperature (°C)	36.3–37.2	37.9	36.7	36.6
Leukocyte count (10^9^/L)	4.0–10.0	10.39	4.98	3.61
Absolute neutrophil count (10^9^/L)	2.0–7.0	9.13	3.51	1.89
Absolute lymphocyte count (10^9^/L)	0.8–4.0	0.49	0.72	0.94
Absolute monocyte count (10^9^/L)	0.12–1.00	0.72	0.63	0.73
C-reactive protein (mg/L)	0.00–8.00	170.91	22.49	15.17
Procalcitonin (ng/mL)	0.00–0.50	0.44	0.12	–
Creatinine (μmol/L)	57–111	82	68	91
Urea (μmol/L)	3.60–9.50	9.12	4.00	5.03
Case2				
Body temperature (°C)	36.3–37.2	39.0	37.8	37.2
Leukocyte count (10^9^/L)	4.0–10.0	38.63	13.7	5.04
Absolute neutrophil count (10^9^/L)	2.0–7.0	34.34	10.32	2.75
Absolute lymphocyte count (10^9^/L)	0.8–4.0	0.97	1.82	1.23
Absolute monocyte count (10^9^/L)	0.12–1.00	3.17	1.33	0.69
C-reactive protein (mg/L)	0.00–8.00	153.19	72.94	17.47
Procalcitonin (ng/mL)	0.00–0.50	6.02	0.34	0.12
Creatinine (μmol/L)	57–97	459	106	84
Urea (μmol/L)	3.10–8.00	37.38	7.53	3.98

### 2.2 Case 2

A 51-year-old immunocompetent man presented to the hospital with sudden onset of left shoulder pain 3 d prior to presentation. The patient was diagnosed with a DeBakey grade Is aortic dissecting aneurysm using aorta CT angiography, and the right renal artery was involved. The patient underwent surgical replacement of the total aortic arch and ascending aorta and stented elephant trunk implantation after completing the preoperative examination. The operation was successful; cefoperazone sodium and sulbactam sodium (4 g divided into two groups) were administered during this stage, and drainage tubes were placed in the pericardium, mediastinum, and left thorax.

Over the next POD, the patient developed low blood pressure, unconsciousness and high fever (39.5 °C), and showed increased inflammatory parameters (leucocytosis 23.53 × 10^9^/L, procalcitonin (PCT) 17.3 mg/L, and C-reactive protein (CRP) 76.20 mg/L). vancomycin (1.0 g every 12 h) and meropenem (1.0 g every 8 h) were empirically introduced. However, culture results for blood, urine, and cerebrospinal fluid were negative. The patient had persistent low-grade fever, impaired liver and renal function, and decreased urination. Continuous Renal Replacement Therapy and other supportive symptomatic treatments were administered. The patient continued to have low fever during this period. On POD 10, a routine urine test and urine culture were performed, and the culture result of the urine sample was negative; however, the routine urine test showed positive leukocyte esterase (++) and a few bacteria. Subsequently, the urinary catheter was replaced.

On POD 11, the patient regained consciousness, although fever persisted (maximum 39.0 °C), and turbid fluid was secreted from the mediastinal area. Mediastinitis was suspected, and mediastinal fluid samples were submitted to the bacteriology laboratory for culture. Linezolid was added (oral, 600 mg twice daily) to the antimicrobial therapy. On POD 17, cultures of the mediastinal fluid and MALDI-TOF MS analysis were positive for *M. hominis*, and the previous antibiotic treatment was substituted with meropenem (0.5 g every 8 h) and moxifloxacin (400 mg) every day. However, the antimicrobial therapy progressed slowly. On POD 31, the patient had an infection of the mid-chest incision, local skin redness, and swelling, despite CT exhibiting an increasing bilateral pleural effusion and the relevant blood test showed CRP 100.30 mg/L. In addition, the patient's clinical condition deteriorated, and developed fever again, requiring operative re-exploration of the mediastinum. The sternotomy site was reopened, aggressive debridement of the infected and necrotic tissues was performed, the sternum was reclosed with steel wires, and mediastinal, thoracic, and subcutaneous drainage tubes were placed. The necrotic tissue sample was subjected to mNGS, which also yielded positive results for *M. hominis*. The antimicrobial was changed to omadacycline (100 mg every day) and josamycin (oral, 400 mg every 8 h), and the patient's liver and kidney functions recovered on the 6th day after operative re-exploration. In subsequent days, the fever gradually subsided, the turbid discharge from the mediastinum stopped, and the drainage tubes were gradually removed. All cultures were negative for *M. hominis* after 10 days of omadacycline treatment. On POD 61, the patient had recovered after 41 days of antimicrobial therapy with moxifloxacin, omadacycline (22 days), and josamycin. The patient was discharged and josamycin (oral, 800 mg daily) was continued for another 12 months.

### 2.3 Literature review

A search of the PubMed and MEDLINE databases was performed until 13 September 2023 including studies written in English or Spanish. The search terms were: “*Mycoplasma hominis* and mediastinitis” (20 results) and “*Mycoplasma hominis* and cardiovascular infection” (25 results). The following criteria were used for inclusion: 1) the reports on cases had a clear confirmed diagnosis, and mediastinitis infection of *M*. *hominis* was confirmed by standard methods; 2) the type of surgery did not include any lung transplantation or heart transplantation; 3) the patients were adults and immunity was normal, excluding those with diseases with compromised immunity, immunosuppressive medication therapy, and autoimmune diseases; and 4) the patient had no other co-associated infection in the mediastinum.

To date, limited cases have been reported in the literature, with most patients presenting with identifiable factors that predispose them to infections with *M*. *hominis* such as immunosuppression or organ transplantation. The final review included nine eligible studies that described 10 cases [5,[7], [8], [9], [10], [11], [12], [13], [14]]. The case reported here has also been included in the literature analysis and review. The data summarised in Table 2 show that almost all patients were men among the 12 cases in which sex was mentioned, including our patient. The median age was 59 years (ranging from 37 to 78 years), and two them were younger than 50 years, and the others were all older than 52 years. Of the 12 patients, six had a history of hypertension, and two were smokers. The types of surgery performed included aortic or valve replacement surgery (7/12, 58.3 %), coronary artery bypass surgery (4/12, 33.3 %), and aortic valve-sparing surgery (1/12, 8.3 %). The median onset of clinical symptoms time was 14.5 d after surgery (ranging from 6 to 26 days). The most frequent clinical signs and symptoms included fever (11/12, 91.7 %), pain (4/12, 33.3 %), sternal dehiscence (3/12, 25.0 %), pericardial or pleural effusion (4/12, 33.3 %), and purulent discharge (7/12, 58.3 %). The mean peak of temperature was 38.5 ± 0.8 °C. Culture and molecular diagnosis play essential roles in the diagnosis of *M*. *hominis.* Culture specimens were used to identify the specific pathogen in almost all cases (11/12), and seven cases were identified using molecular diagnosis. The positive time for *M. hominis* separation or detection was more than a few days after symptom onset. Empirical treatments barely covered *M. hominis*, and targeted treatments mainly included levofloxacin, doxycycline, clindamycin, and omadacycline. Regarding the outcomes of these reviewed cases, two patients died, and 10 survived and recovered. The targeted treatment duration ranged from 3 weeks to 16 months in surviving patients.

**Table 2:** Summary of data on previously reported cases of mediastinitis caused by *Mycoplasma hominis*.

Cases	Reference	Age (years), Sex	Country	Previous Disease(s) and Predisposing Factor(s)	Diagnosis	Surgery	Main Symptom(s)	Infection Time (POD)	Species Isolated (Method) and Positive time	Positive Sample	Empirical Treatment	Targeted Treatment (Duration)	outcome
1	2014 [7]	37, Male	France	Hypertension and tobacco smoking	Type A aortic dissection	Ascending aorta replacement	Fever and purulent discharge	POD 20	MH (16 S rDNA secquece), POD 27	Sternal bone and pre-sternal abscess biopsies	PTZ, DAP and CAS	LEV (6 months)	Survive
2	2021 [8]	54, Male	Japen	Ménierè syndrome	Aortic valve stenosis and patent foramen ovale (PFO)	Aortic valve replacement and patent foramen ovale closure	Fever, pain and purulent discharge	POD 15	MH (culture and PCR), POD 18	Abscess and tissue samples	SAM, MEM and VA	LEV and MIN for 3 weeks	Survive
3	2010 [9]	55, Male	Switzerland	Hypertension	Acute type A aortic dissection	Ascending aorta surgical replacement	Sternal dehiscence, pericardial and bilateral pleural effusion	POD 22	MH (culture and PCR), POD 44	Pericardial discharge, sternum and aortic graft	IMP, VA and PTZ	DOX (4 months) and MXF (16 months)	Survive
4	2008 [10]	77, Male	Spain	Hypertension and chronic obstructive pulmonary disease	NM	Aortic valve and root replacement	Fever, sternal dehiscence and mediastinal purulent fluid	POD 14	MH and UU (culture and 16 S rRNA sequence), POD 24	Subcutaneous tissue, muscle and synthetic material	CTX, MNZ, PTZ and TEC	DOX and CLI (several days)	Death
5	2020 [11]	63, Male	Slovenia	Arterial hypertension	Ascending aortic aneurysm	Aortic valve sparing surgery	Sepsis, spiking fever and pericardial effusion	POD 16	MH (culture and RTPCR), POD 23	Blood	IMP-cilastatin and VA	TGC and LEV (8 days), DOX (12 months)	Survive
6	2014 [12]	56, NM	Switzerland	NM	Acute type A aortic dissection	Ascending aorta replacement	Fever and purulent discharge	POD 26	MH (culture), POD 29	Mediastinal tissue, sternum and aortic prosthesis	IMP and VA	Vibramycin and Avalox (5 months)	Survive
7	1996 [13]	62, Male	the Unite States	NM	Triple-vessel coronary artery disease	Coronary artery bypass surgery	Fever, pain and purulent discharge	POD 17	MH (culture), POD 23	Mediastinal tissue	CFM	DOX (4 weeks)	Survive
8	1999 [5]	78, Male	Finland	Heart infarction	Cardiac failure	Coronary artery bypass surgery	Fever, pain and purulent discharge	POD 6	MH (culture), NM	Pleural fluid	IMP, VA, ERY and FCA	CLI, CIP, RIF and FCA (NM)	Survive
9	1999 [5]	68, Male	Finland	Smoke and hypertension history	Cardiac failure	Coronary artery bypass surgery	Fever, pain and purulent discharge	POD 12	MH (culture), NM	Sternum wound specimens	IMP, VAF, ERY and FCA	CLI (NM)	Death
10	1987 [14]	48, Male	the Unite States	NM	Unstable angina	Coronary artery bypass surgery	Fever and purulent effusion	POD 6	MH (culture), NM	Sternal bone tissue and mediastinal and left pleural fluid samples	TOB, VA and TIC	DOX, CLI and TOB (NM)	Survive
11	PR	71, Male	China	Hypertension	Acute type A aortic dissection	Total aortic arch and ascending aorta surgical replacement	Fever, sternum unstable and pericardial and right pleural effusion	POD 14	MH (culture and mNGS), POD19	Pericardial and pleural discharge	CXM，MEM	Omadacycline (52 days) and LEV (56 days)	Survive
12	PR	52, Male	China	/	Acute type A aortic dissection	total aortic arch and ascending aorta surgical replacement,	Fever, pericardial effusion and bilateral pleural effusion	POD 11	MH (culture and mNGS), POD17	mediastinal purulent fluid	CSL, VA, MEM	MFX, omadacycline and JOS (41 days) and JOS (12 months)	Survive

PR present report, POD postoperative day, NM Not mentioned, MH *Mycoplasma hominis,* UU *Ureaplasma urealyticum,* PTZ piperacillin-tazobactam, DAP daptomycin, CAS caspofungin, LEV levofloxacin, SAM sulbactam-ampicillin, MEM meropenem, VA vancomycin, MIN minocycline, IMP imipenem, DOX doxycycline, MXF moxifloxacine, CTX cefotaxime, MNZ metronidazole, TEC teicoplanin, CLI clindamycin, TGC tigecycline, CFM cefamandole, ERY erythromycin, FCA fluconazole, CIP ciprofloxacin, RIF rifampin, TOB tobramycin, TIC ticarcillin, CXM cefuroxime sodium, CSL Cefoperazone-sulbactam, MFX moxifloxacine, JOS josamycin.

### 2.4 Statistical analysis

All data were analysed using the SPSS statistical software package (version 19; IBM SPSS Statistics). Data of normal distribution were expressed as the mean ± standard deviation (x ± s), and data of abnormal distribution by median and range. The collected count data were evaluated using descriptive statistics, including numbers and percentages.

## 3 Discussion

*Mycoplasma hominis* is typically identified as a genitourinary pathogen, and the colonisation rates of *M. hominis* in the genitourinary system range from 21 % to 53 % worldwide [1]. Owing to advances in diagnostic techniques, surgical infections caused by *M. hominis* have been reported, particularly in immunocompromised patients or patients undergoing organ transplantation [15]. Here, we report two rare cases of mediastinitis caused by *M. hominis* after cardiac surgery in two immunocompetent patients and review previous reports on mediastinitis caused by *M. hominis.* Patients with lung or heart transplantation-associated *M. hominis* infections were excluded from the study. All cases of mediastinitis caused by *M. hominis,* including the two reported here, were postcardiac. No case of non-postoperative or other post-operative mediastinitis caused by *M. hominis* was reported. The median age of the 12 patients was 59 years, and almost all patients infected with *M. hominis* were men. This can be partly explained by the fact that patients undergoing cardiac surgery were predominantly men (72 %) [16]. Although the origin of the infection is difficult to identify, it can be hypothesised that postoperative *M. hominis* mediastinitis is linked to urinary catheterisation. Urinary catheterisation is more traumatic in men, and may lead to the implantation of *M. hominis* at the surgical site [13]. Indeed, two patients in this study underwent secondary urinary catheterisation, which has also been mentioned in the reviewed literature and other reports as a possible cause of postoperative *M. hominis* infection [7,8,11]. Catheterisation is a common procedure during surgery, and the possibility of postoperative *M. hominis* infection is often underestimated.

**Fig. 1:**
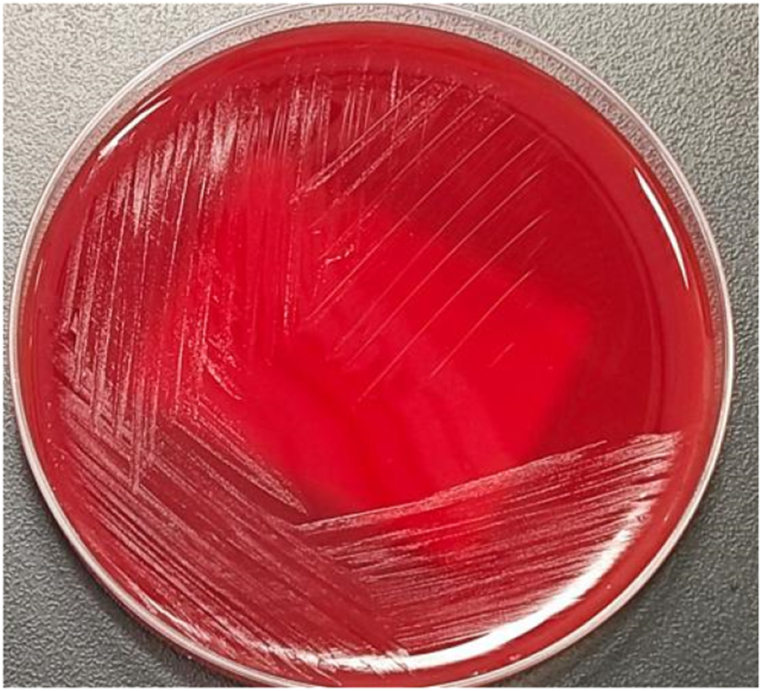
Characteristics of the *Mycoplasma hominis* colonies on columbia blood agar.

The diagnosis of extragenital *M. hominis* infections is often challenging because of the fastidious and slow growth of colonies and the absence of a cell wall, which is not visible using Gram staining. Culture is considered the gold standard for the detection of *M. hominis*. *M. hominis* can form typical fried-egg colonies on agar and is less fastidious than other *Mycoplasma* spp [1]. A generic medium, such as Columbia blood agar, supports growth if incubation is prolonged. Very tiny colonies of *M. hominis* (0.2 mm) appeared on days 2–7 when the Columbia blood agar medium was incubated under 5 % CO_2_ or anaerobic conditions (Fig. 1). As a result, it would easily be missed by such an approach unless *M. hominis* infection was considered in advance. However, many cardiovascular surgeons are unaware that *M. hominis* can cause postoperative infections, especially as blood cultures are usually negative. The inability to detect the growth of these organisms using automated blood cultures has been attributed to a great extent to the static mycoplasma effects of sodium polyanethol sulphonate, an anticoagulant widely used in liquid blood culture media [17]. This represents a major impediment to its identification using standard culture media and explains the delay in correct diagnosis and treatment in the reviewed cases. Molecular methods represent attractive options for diagnosing *M. hominis* infections. Previous reports have shown that 16 S rDNA sequencing taken directly from clinical samples is a good method for identifying *M. hominis*, and PCR techniques targeting *M. hominis*-specific genes can detect as few as 7 × 10^3^ copies/mL [7,8]. These techniques are more sensitive than culturing clinical samples. As shown in Table 2, 16 S rDNA sequences and PCR were used for cases 1, 4 and 2, 3, and 5. In our study, attentive microbiology laboratory staff found and reported the results of *M. hominis* culture and MALDI-TOF MS analysis. As our patient was critically ill, mNGS was used, and positive results were reported. Metagenomic next-generation sequencing (mNGS) technologies have emerged as powerful tools for molecular biology research and clinical diagnostic applications [18]. Disease diagnostics, pathogen detection, and genetic mutation detection are growing clinical areas in which mNGS is widely used. We suggest that molecular methods such as mNGS should be used in cases of culture-negative samples or unusual culture results for patients presenting clinical symptoms of mediastinitis in order to be able to rapidly detect a broad-range of pathogenic bacteria, including *M. hominis*.

Beta-lactam antibiotics, such as cefazolin and cefuroxime, and glycopeptides, such as vancomycin, are typically used for antimicrobial prophylaxis in cardiac surgery or for the treatment of postoperative infections after cardiac surgery, targeting Gram-positive cocci, including *staphylococcus aureus* and Gram-negative bacilli. However, these molecules are not active against *M. hominis* because the lack of a cell wall renders *Mycoplasma* non-susceptible to the action of beta-lactam antibiotics. Empiric antibiotic therapy has invariably been directed toward common Gram-positive pathogens. No patient had received agents active against *M. hominis* before the culture, and the molecular results revealed the presence of this pathogen (Table 2). *M. hominis* is generally susceptible to tetracyclines, clindamycin, and fluoroquinolones but is intrinsically resistant to clarithromycin and erythromycin. However, resistance to fluoroquinolones, tetracyclines, and clindamycin of *M. hominis* varies by report [6]. Our patient responded rapidly to omadacycline, which is effective against *M. hominis*, allowing sternal closure without resorting to an omental flap, given the excellent clinical response to antibiotics, followed by rapid wound healing in Case 1. However, in Case 2, failure to clear the infection with moxifloxacin therapeutically and the patient received omadacycline.

Omadacycline (9-neopentylaminomethylminocycline) is a novel and promising aminomethylcycline available in both intravenous and oral formulations. Omadacycline had the lowest MIC_90_ among all drugs tested against *M. hominis* and its activity was not affected by macrolide, tetracycline, or fluoroquinolone resistance [19]. In addition, patients with hepatic and renal insufficiency, including those with end-stage renal disease undergoing haemodialysis, do not require omadacycline dose adjustments [20]. To the best of our knowledge, this is the first report of the successful treatment of mediastinitis caused by *M. hominis* using omadacycline.

The median onset of clinical symptoms was 14.5 d after surgery, and the time of *M. hominis* separation or detection was more than a few days after symptom onset (Table 2). The initial treatment for mediastinitis includes aggressive debridement of infected and necrotic sternal tissues, followed by drainage and specific antimicrobial therapy. Signs of infection are usually not evident early postoperatively, and the incision or skin status during surgery is usually not significantly abnormal. In some reviewed cases, the infection followed a localised chronic course with low-grade fever but without septic symptoms. In our patient, the infection was characterised by a chronic and indolent course, which is consistent with other reports. After major surgery, patients may experience other symptoms that often interfere with early diagnosis. Other Mycoplasma species have also been observed in infections involving the mediastinum. *Ureaplasma urealyticum* infrequently coexists with *M. hominis* (case 4) [10].

The ideal duration of antibiotic treatment is unknown, and long-term suppressive therapy with antimicrobials is always recommended due to the presence of heart implants. In this study, the targeted treatment duration ranged from 3 to 16 months in the surviving patients. Two of the 12 patients described to date died due to uncontrolled *M. hominis* infection, and our two patients had a good prognosis with targeted therapy. Therefore, careful observation of the clinical course after the administration of antimicrobial agents and extension of the duration of treatment for *M. hominis* are needed.

In summary, we identified two cases of postoperative *M. hominis* mediastinitis in immunocompetent patients and reviewed 10 other cases previously described in the literature. Therefore, the role of *M. hominis* as a cause of postoperative infection during cardiac surgery may be underestimated. This infection should be included in the differential diagnosis in conjunction with chest imaging features if the patient has a persistently low fever and negative blood culture. When a case is suspected, clinical bacteriological laboratories and infectious disease experts should be consulted for the isolation of *M. hominis* and empirical antibiotic treatment against mycoplasmas should be considered. Therefore, molecular diagnostics should be considered more frequently in such cases. A preoperative urine cultures can be considered in patients with renal insufficiency, multiple catheterisations, abnormal routine urine, or urinary tract infections. Currently, there are no guidelines for the management of *M. hominis* mediastinitis. Thus, reporting new cases is useful for sharing successful treatment strategies.

## CRediT authorship contribution statement

**Fang Wang:** Writing – original draft, Methodology, Investigation, Data curation. **Qing Zhan:** Writing – original draft, Investigation, Data curation. **Anfeng Yu:** Methodology, Investigation. **Hongchao Chen:** Methodology. **Yan Zhang:** Methodology, Funding acquisition. **Qing Yang:** Investigation, Data curation. **Tingting Qu:** Writing – review & editing, Methodology, Conceptualization.

## Ethical approval and consent to participate

Written informed consent was obtained from all the patients involved in the publication of any potentially identifiable images or data included in this article. This study was approved by the local ethics committee of the First Affiliated Hospital, College of Medicine, Zhejiang University (approval number 2024-0390, on 12 April 2024).

## Data availability statement

The original contributions of this study are included in the article/supplementary material section. Further enquiries can be directed to the corresponding authors.

## Funding

This work was funded by the 10.13039/501100012166National Key Research and Development Program of China (2021YFC2301800) and Scientific Research Fund of the Zhejiang Provincial Education Department (Y202146838).

## Declaration of competing interest

The authors declare that they have no known competing financial interests or personal relationships that could have appeared to influence the work reported in this paper.
