# Supplementary material for: Mediastinitis caused by Mycoplasma hominis in immunocompetent patients: A case series report and literature review
Source: Heliyon. 2024 Oct 24;10(22):e39763. doi: 10.1016/j.heliyon.2024.e39763 (PMC11617930; doi:10.1016/j.heliyon.2024.e39763)
Supplement: Multimedia component 1 [file mmc1.docx]

**Metagenomic next-generation sequencing (mNGS) assay**

DNA-based mNGS testing (shotgun sequencing) for whole blood samples of each patient was performed in Department of Laboratory Medicine. Firstly, the plasma was extracted from whole blood samples. According to the manufacturer's instructions, the DNA were extracted from plasma samples using the QIAamp® UCp Pathogen DNA Kit (Qiagen, Duesseldorf, Germany). Human DNA was removed using 1U Benzonase (Sigma) and 0.5% Tween 20 (Sigma) and incubated at 37℃ for 5 min. The extracted DNA was then quantified using a Qubit dsDNA HS Assay Kit (Invitrogen, Carlsbad, USA). Then, the quantified unique DNA fragments were spiked for each sample as an identity and internal control, which were PCR products of Oryza sativa 400 to 600 bp in length. The eluate were used to generate libraries using the Nextera DNA Flex kit (illumina, San Diego, USA). Library pools were then loaded onto the illumina Nextseq CN500 sequencer for 50 cycles of single-end sequencing (SE-50), generating approximately 20 million reads for each library. The sequence data was analyzed by using Weiyuan-MG mNGSsoftware (Weiyuan, Weiyuan Biotechnology, China) which contains a proprietary curated database consisting of morethan 20,000 reference microbial genomes. The analyzed data were then reported for species and relative abundance of pathogens

**The culture and identification method for** ***M. hominis***

The samples were inoculated on Colombia blood agar and then the Columbia blood agar medium is incubated in 37 ℃ and 5% CO2. According to the manufacturer's instructions, the isolate was identified by by matrix-assisted laser desorption ionization timeof-flight mass spectrometry (MALDI-TOF MS).
